# Effect of Protein-Glutaminase on Calcium Sulphate-Induced Gels of SPI with Different Thermal Treatments

**DOI:** 10.3390/molecules28041752

**Published:** 2023-02-12

**Authors:** Xin Li, Liwei Fu, Zhiyong He, Maomao Zeng, Qiuming Chen, Fang Qin, Zhaojun Wang, Jie Chen

**Affiliations:** 1 State Key Laboratory of Food Science and Technology, Jiangnan University, Wuxi 214122, China; 2 International Joint Laboratory on Food Safety, Jiangnan University, Wuxi 214122, China

**Keywords:** soy protein isolates, protein-glutaminase, calcium sulphate-induced gels, aggregation

## Abstract

The effects of protein-glutaminase (PG) on calcium sulphate (CaSO_4_)-induced gels of soy protein isolate (SPI) with different heat treatment levels were investigated. The time-dependent degree of deamidation showed that the mild denaturation of the protein favored the deamidation. The particle size distribution showed that the heat treatment increased the SPI particle size, and the particle size distribution of the SPI shifted to the right or increased the proportion of the large particle size component as the degree of deamidation increased for each sample. Rheological analysis showed that the deamidation substantially pushed up the gel temperature and decreased the value of G′. The gel strength and water-holding capacity showed that the higher the amount of enzyme added, the more significant the decrease in gel strength, while the gel water-holding capacity increased. In summary, the deamidation of PG and heat treatment can affect the gel properties of SPI synergistically.

## 1. Introduction

Soybean protein isolate (SPI) has become a widely used food ingredient because of its relatively low price, high nutritional value and good functional properties [1]. The gelation of SPI is one of the most important functional properties, both in the traditional applications of soy [2] and meat products [3] and in the emerging applications of plant simulants [4], plant eggs [5], etc. The current gelation methods of soy protein are divided into heat-induced gels and cold-set gels, and cold-set gels include acid-induced gels, salt-induced gels and enzyme-induced gels [6,7]. As a cold-set gelation method, salt-induced gelation can avoid the loss of some heat-damaged flavor components or bioactive components during the gelation process, which has generated a lot of interest [8].

Salt-induced SPI gelation can be achieved by adding salt coagulant to a solution of preheated denatured SPI (the concentration of SPI is below the critical concentration for thermal gelation) and allowing it to gel [9]. During the gelation process, the salt coagulant can dissociate and release Ca^2+^, Mg^2+^, etc. These ions can shield the negative charge on the surface of protein molecules and can exchange with H^+^ on the carboxyl group, forming salt bridges [10]. Soy proteins form a gel network structure through electrostatic interactions, hydrophobic aggregation and linkage of calcium bridges. The current study shows that the type of salt ions [11], their concentration [12], the combination of different salt ions [13], the concentration of SPI [14] and the degree of denaturation and aggregation of SPI [15] all affect the properties of the final salt-promoted gels. Compared with other cold-set gels, salt-induced gels had stronger texture without sour taste [15]. However, the salt-induced gels with CaSO_4_, CaCl_2_, MgCl_2_, etc. often suffer from insufficient water-holding capacity [13,16]. In general, the water-holding capacity of heat-induced gels is in the range of 85%~90% [17], and those of acid-induced gels are between 70% to 85% [18], and the enzyme-induced gels were in the range of 60%~90% [19]. In contrast, calcium-induced protein gels ranged from about 50% to 90% [20]; calcium-induced SPI emulsion gels were only in the range of 50~75% [13]. In fact, a large number of studies have focused on how to improve the gel strength of salt-induced gels [21,22,23], but the improvement of the water-holding capacity of salt-induced gels has been reported relatively rarely.

Water-holding capacity (WHC) reflects the ability of a gel to effectively immobilize water within its matrices through capillary force, which is one of the important indicators for evaluating a gel [24]. Salt-induced gel properties are highly dependent on the type and amount of salt ions [12,25], protein concentration [14], as well as the degree of protein denaturation and aggregation [15]. Among the coagulant types of CaSO_4_, CaCl_2_ and MgCl_2_, CaSO_4_-induced gels have better water-holding capacity than CaCl_2_-induced and MgCl_2_-induced ones, and the former can form relatively homogeneous gels [13]. The gel structure formed by increased salt ion strength also becomes rough and inhomogeneous [26]. The effect of the type and concentration of ions on gel strength and water-holding capacity is related to the gelling speed and the structural properties of salt-induced gels, with too fast a gel speed and too rough a structure being detrimental to gel water-holding capacity [27,28]. From a protein perspective, higher protein concentrations produce stiffer gels with better water-holding capacity, due to more protein molecules being cross-linked and involved in the formation of network structures. The water-holding capacity of salt-induced gels is also related to the size of the aggregates formed during the SPI preheating treatment [29,30]. Zhao et al. showed that the water-holding capacity of gels is strongly related to the degree of denseness of the gel network. Thermal denaturation significantly increased the surface hydrophobicity of proteins and promoted the aggregation of protein molecules with different degrees of hydrophobicity, forming a series of aggregates with different particle sizes [15]. During the gelation process, large aggregates first aggregate to form the basic network structure of the gel, while small aggregates further bond to the network to form the final gel structure. Wang et al. showed that the size and number of protein aggregates had an important effect on the salt-induced gel properties, and that large and numerous protein aggregates were beneficial for enhancing the strength and water-holding capacity of calcium-induced soy protein emulsion gels [29]. Therefore, it can be hypothesized that for salt-induced gels, controlling the heat-induced structural unfolding of SPI and the resulting aggregation size, controlling the surface hydrophobicity of the aggregated, and thus the gelation rate will contribute to the formation of a homogeneous gel network.

Protein-glutaminase (PG) is an enzyme that catalyzes the deamidation of glutamine residues in proteins which can be used to reduce surface hydrophobicity and thus increase protein solubility [31]. It was found that in the process of deamidation, on the one hand, protein de-folding leads to conformational changes, exposing the hydrophobic groups embedded inside the molecule and increasing the value of surface hydrophobicity; on the other hand, deamidation converts amide groups to carboxyl groups, increasing negative charges and enhancing the electrostatic repulsion between protein molecules, which improves solubility [32]. Therefore, it can be further hypothesized that the changes in conformation and hydrophobic region distribution due to deamidation would change the salt-induced gelation rate of the protein, which in turn would improve the water retention of the salt-induced gel.

This paper focuses on testing the above hypothesis that adding PG to SPI with different degrees of thermal denaturation changes the soy protein aggregate size and surface hydrophobicity, which could consequently change the speed and structure of CaSO_4_-induced gels and improve their water-holding capacity.

## 2. Results

### 2.1. Degree of Deamidation

The deamidation of the PG on the glutamine group in the side chain of a protein or long peptide produces glutamate and releases ammonia, so the process of the enzyme reaction can be monitored by measuring the amount of ammonia [33].

The degree of deamidation of all samples increased with time, the reaction was rapid in the first 1 h and the degree of deamidation increased rapidly. After 2 h, the reaction rate gradually slowed down, possibly due to the PG deamidation reaction approaching saturation as time increased [34]. The n-5U reached 33% deamidation after 9 h of reaction, which is closer to the reported results [35].

**Figure 1:**
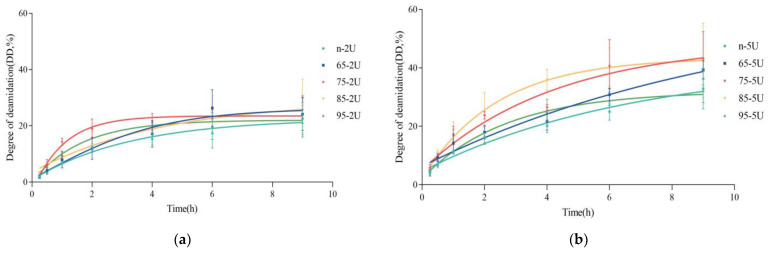
The degree of deamidation (DD, %) of SPI induced by protein-glutaminase (PG) deamidation with time reaction. (**a**): addition of 2 U/g PG; (**b**): addition of 5 U/g PG.

The temperature of preheat treatment had a significant effect on the deamidation reaction of SPI. As seen in Figure 1, at the addition of 2 U/g PG, the deamidation reaction process was slightly accelerated in the 65 °C preheated treated samples, and the final deamidation degree was also improved compared to the control. 75 °C preheated treated samples had a much faster deamidation reaction than those at 65 °C. 85 °C and 95 °C preheated treated samples had a slightly faster deamidation reaction process in the first half hour than 75 °C, but after half an hour, it became slower and the final degree of deamidation was comparable to that of the 65 °C and 75 °C.

At the addition of 5 U/g PG, the SPI pre-heated at 75 °C and 85 °C had the fastest deamidation reaction process and the highest degree of deamidation, followed by the sample pre-heated at 65 °C. In contrast, the deamidation process and the final degree of deamidation of the samples at 95 °C were almost comparable to the control. The overall deamidation process and the final degree of deamidation were higher with adding 5 U/g PG than 2 U/g.

The effect of preheat treatment temperature on the deamidation reaction and the final degree of deamidation of SPI may be related to the degree of structure unfolding and protein–protein aggregation after preheating treatment. The denaturation temperatures of SPI ranged from 70 °C to 75 °C for 7S and 90 °C to 95 °C for 11S [36]. At 65 °C the 7S structure partially unfolded while the 11S was not. At 75 °C the 7S was totally denatured, while the 11S partially unfolded. Treatment at 85 °C allows the 7S to be fully denatured, the 11S structure to unfold relatively fully, and the system started to aggregate. At 95 °C, the SPI was heavily denatured and caused relatively serious heat aggregation [37]. The deamidation progression of NSPI and four different degrees of denaturation of soy protein implied that the mild denaturation and structural unfolding of 7S or 11S, where the molecular structure becomes more flexible in solution and more amide-group interaction sites are exposed, may be favorable for the deamidation of PG. In contrast, full denaturation may limit the PG deamidation reaction due to severe thermal aggregation and greater molecular stiffness. Jiang et al. also found that the degree of deamidation of the preheated SPI at 100 °C and 121 °C was lower than that of the control sample [32].

### 2.2. Particle Size Distribution

**Figure 2:**
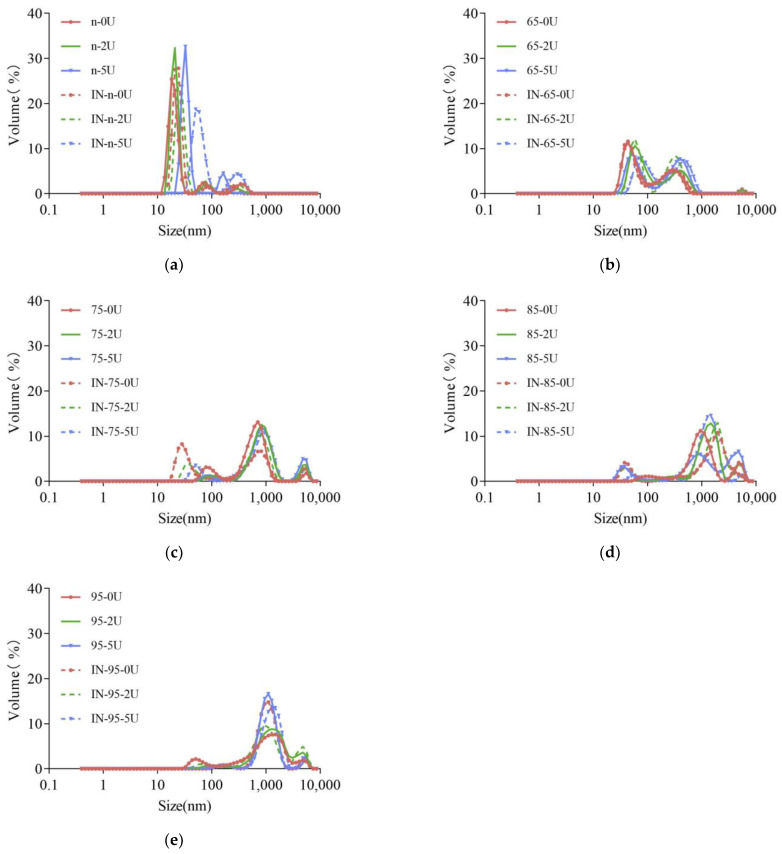
Particle size distribution of SPI samples. Samples after inactivating the PG named IN-n-0U, IN-n-2U, IN-n-5U, IN-65-0U, IN-65-2U, IN-65-5U, IN-75-0U, IN-75-2U, IN-75-5U, IN-85-0U, IN-85-2U, IN-85-5U, IN-95-0U, IN-95-2U, IN-95-5U, respectively. (**a**): native SPI with different PG-treated levels; (**b**): preheated SPI at 65 °C with different PG-treated levels; (**c**): preheated SPI at 75 °C with different PG-treated levels; (**d**): preheated SPI at 85 °C with different PG-treated levels; (**e**): preheated SPI at 95 °C with different PG-treated levels.

The overall particle size distribution of the SPI solution shifted to the right as the heat treatment intensity increased (Figure 2), indicating that the heat treatment caused the system to form larger aggregates. This is consistent with previous studies. Wang et al. [29] also found that the SPI had a more spacious structure and larger particle size with higher heating temperatures.

After deamidation of SPI at each preheat treatment temperature, the overall particle size distribution of SPI solution shifted to the right, or the percentage of large size increased with the increase of deamidation degree for each sample except for Native. Further heating of the enzyme-treated sample to inactivate the enzyme would further trigger a continued shift of the particle size distribution to the right or a continued increase in the percentage of large particle size. The above results indicated that deamidation could lead to structural unfolding and increase protein aggregation. This structural unfolding can further lead to aggregation upon subsequent heating. Jiang et al. [32] also found that the PG deamidation reaction promoted the formation of more protein aggregates by SPI sulfhydryl oxidation or sulfhydryl–disulfide bond interconversion. 

The changes in molecular weight and the aggregation of different proteins after deamidation were different. Jiang et al. [32] also found that SPI deamidation further enhanced the aggregate content. Jiang et al. [34] showed insignificant changes in the relative molecular mass distribution of the products after PG deamidation of oat protein. Suppavorasatit et al. [35] found an increase in the proportion of small molecules in commercial soy proteins after deamidation, which the authors attributed to the high content of protein hydrolases in PG. Yong [38] found that the relative molecular mass of gluten protein increased with PG deamidation, which the authors attributed to the increased electrostatic repulsion within the molecule. Zhao et al. [39] found that barley gluten may have produced disulfide-linked aggregates after deamidation. The effect of PG on the solubility and the number of aggregate sizes of proteins may be related to the fact that the deamidation process triggers different structural unfolding and aggregation forces. 

### 2.3. Surface Hydrophobicity

**Figure 3:**
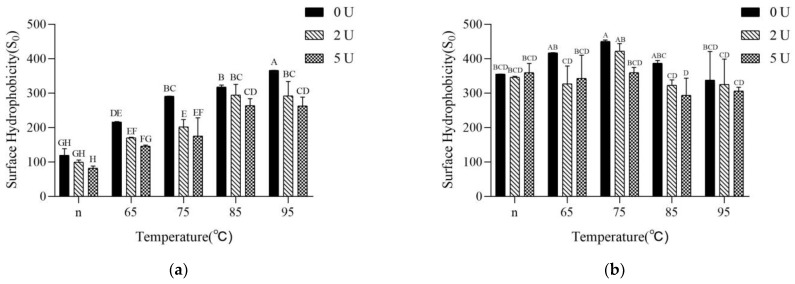
Surface hydrophobicity of the SPI samples. (**a**): before inactivating the PG; (**b**): after inactivating the PG. Different capital letters indicate significant differences (*p* < 0.05).

In order to verify the reasons for the changes in particle size distribution by heating and deamidation, the S_0_ of each protein sample after deamidation was measured before and after terminating the enzymatic reaction. The S_0_ of SPI increases significantly after heat treatment (Figure 3), which is in agreement with the results of previous studies [32]. The S_0_ of the protein decreased slightly after PG treatment, and the degree of decrease in S_0_ increased with the increase of deamidation (Figure 3a). It is noteworthy that the degree of decrease in S_0_ is the greatest for the samples preheated at 75 °C, which is consistent with the data that the degree of deamidation is the greatest for the samples preheated at 75 °C (Figure 1). This result combined with the particle size results also further indicates that light preheating treatment, which makes 7S denaturation or 11S lightly unfolded, is favorable for deamidation, while severe treatment, where the degree of aggregation becomes larger and the structure becomes relatively rigid, is unfavorable for deamidation.

After terminating the enzymatic reaction, the S_0_ increased further for the four groups of samples except for the 95 °C preheat-treated samples, especially the three groups of native, 65 °C and 75 °C preheat-treated samples increased very significantly. At the same time, the inactivation treatment made the difference of S_0_ before and after the deamidation of the samples after various preheating treatments smaller than before inactivation (Figure 3b).

### 2.4. Rheological

Storage modulus (G′) is an indicator of the energy reserved every cycle of deformation, and its value reflects the elastic or solid-like nature of the specimen being tested, while the loss modulus (G″) is a measure of the energy dissipated by the specimen and is related to the viscous or liquid-like nature of the specimen [40].

Compared with the rheological curves of the samples with different heating levels after calcium addition, it can be found that the gel temperature of CaSO_4_-induced gels decreased rapidly with the increase of preheating treatment, while the final G′ of gels increased rapidly (Table 1). This could be related to the formation of aggregates by pretreatment, since large protein aggregates were more likely to interact with Ca^2+^ than small aggregates. For calcium-promoted emulsion gels, Wang et al. [29] also found that large and numerous protein aggregates contributed to the decrease in emulsion gel temperature and the increase in G′ of the final system.

**Figure 4:**
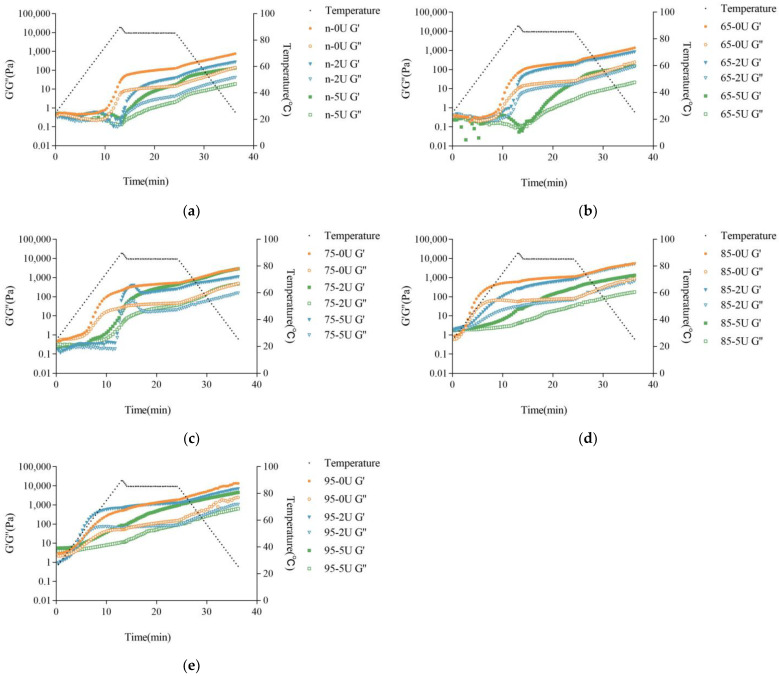
Rheological properties of the soy protein with different heating levels and PG-treated after calcium addition. (**a**): native SPI with different PG-treated levels; (**b**): preheated SPI at 65 °C with different PG-treated levels; (**c**): preheated SPI at 75 °C with different PG-treated levels; (**d**): preheated SPI at 85 °C with different PG-treated levels; (**e**): preheated SPI at 95 °C with different PG-treated levels.

The rheological characteristics of the enzyme-treated system with calcium were significantly different from those of the untreated one and were strongly correlated with the amount of enzyme added and the degree of preheating of the SPI. In the case of 2 U/g of enzyme addition, the gel temperature of NSPI increased and the gel time was delayed after being treated by PG, and the G′ of the final gel decreased. In the 65 °C preheat treatment system, the PG treatment pushed up the gel temperature and caused a mild decrease in the final G′ and G″ values. 75 °C and 85 °C preheat treatment systems pushed up the gel temperature substantially and delayed the gel time but did not affect the final G′ and G″ values compared to the non-enzyme treated samples. The preheated system at 95 °C, with 2 U/g of PG addition, surprisingly lowered the gel temperature, but caused a significant decrease in G′ and G″ values. The curve of G′ of the system with 5U of enzyme addition differed greatly from that of the control without enzyme addition, and all the curves, had the pattern of rising, falling and then rising again, with the most significant one at 75 °C, and the decreasing and a rising trend was relatively insignificant at 85 °C and 95 °C (Figure 4). Moreover, for the systems of native and 65 °C, the first rise had not yet reached the level of gel, and the second rise completed the transition from sol to gel. In contrast, for the systems at 75 °C, 85 °C and 95 °C, the change from sol to gel was completed by the first rise. In addition, for the system with 5 U/g of enzyme addition, the gelation rate was very slow except for the sample at 75 °C.

The effect of PG on the rheological characteristics of the systems after calcium addition and different preheating treatments could be strongly related to the aggregate size and distribution as well as surface hydrophobicity, which resulted from the preheating treatment as well as the PG treatment together. The rheological results, combined with the previous results of deamidation, particle size distribution and surface hydrophobicity, showed that the protein structure unfolding and the larger aggregate size favored the decrease of the temperature of CaSO_4_-induced gels and the increase of G′ and G″ of the final gels, while the increase of deamidation caused the rate of CaSO_4_-induced gels to be significantly slower. However, there was no positive relationship between the deamidation degree, particle size and surface hydrophobicity and the final G′, which implied that the interaction forces of the CaSO_4_-induced gels may be different for the deamidated treated proteins than for the samples not treated with PG. It was generally believed that the decrease in the pH of the solution during CaSO_4_-induced gelation and the binding of Ca^2+^ to protein molecules played an important role [10]. A gradual decrease in solution pH can clearly be observed when Ca^2+^ binds to protein molecules [42]. The decrease in pH weakened the electrostatic interactions between proteins and thus promoted the aggregation of protein molecules. Ca^2+^ achieved the binding of protein molecules by bridging with the carboxyl groups of glutamyl and aspartyl residues and the imidazole groups of histidine residues, and promoted protein molecules to join with each other, leading to gel formation [26]. In contrast, proteins aggregated by preheating can form gels at lower calcium ion concentrations or form gels faster at the same calcium ion concentration compared to native proteins. More and larger aggregates can be favorable for the formation of strong gels [15]. PG delamination would convert the amide group on SPI to the carboxyl group, which introduces a negative charge and can enhance the electrostatic repulsion between protein molecules [43]. Deamidation reduced the number of hydrophobic amino acids, which weakened the surface hydrophobicity of the protein and weakened hydrophobic interaction. Therefore, it can be speculated that the addition of calcium ions to the PG-treated protein system could slow down the CaSO_4_-induced aggregation due to the relatively strong electrostatic interactions, while the increase of carboxyl groups, on the other hand, diminished the efficiency of calcium ions as coagulants and also slowed down the CaSO_4_-induced aggregation and weakened the final gel strength.

### 2.5. Gel Strength and Water-Holding Capacity (WHC)

**Figure 5:**
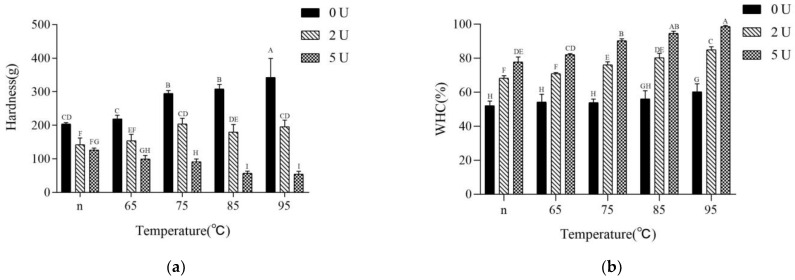
Properties of gels influenced by the different heating levels and PG-treated. (**a**): gel hardness; (**b**): water-holding capacity (WHC). Different capital letters indicate significant differences (*p* < 0.05).

The results of CaSO_4_-induced gel strength and water-holding capacity showed that the gel hardness gradually increased with the increase of pretreatment temperature, from 204 ± 3 g to 343 ± 56 g. However, the water-holding capacity rose only slightly, from 52% to 60% (Figure 5). Hu et al. [20] also found that the water-holding capacity of untreated CaSO_4_-induced soy protein gels was only between 40% and 60%. After PG treatment, the gel strength decreased compared with untreated gels, and the greater the amount of enzyme added, the more significant the decrease in gel strength. However, the water-holding capacity of the gels was significantly improved after PG treatment, and the higher the amount of enzyme added, the higher the improvement of water-holding capacity of the gels.

Comparison of gel strength and water-holding capacity, with the degree of deamidation and rheological properties showed that the water-holding capacity was highly dependent on the gelling speed, and the slower the gelling speed, the higher the gel temperature on the temperature scan curve and the higher the water-holding capacity of the product. If the gelation speed was similar, the higher degree of deamidation and relatively weak hydrophobicity resulted in higher water-holding capacity.

It was generally believed that protein gels with a homogeneous and dense structure have higher gel strength and water-holding capacity than those with an inhomogeneous gel structure [26]. The homogeneity of the gel was related to the speed of gelation and the size and number of aggregates during gelation, with large aggregates tending to form thick gel chains and dense structures [1]. This dense structure also enhanced the strength of the gel while improving water-holding capacity [13]. However, it was observed from the results of this study that the size and number of aggregates certainly affected the gel strength and water-holding capacity, but not in proportion to the strength and water-holding capacity of CaSO_4_-induced gels, and that the degree of deamidation and the rate of gelation all affected the gel strength and water-holding capacity. If the increase in particle size and hydrophobicity was triggered by heating and aggregation, it was significantly favorable for gel strength and also had a slightly positive effect on water-holding capacity. If the larger particle size and hydrophobicity enhancement were caused by unfolding and aggregation of the structure after deamidation, it was favorable for water-holding capacity, but not for gel strength. This result implied that the altered force of CaSO_4_-induced gels could be inherent in the change of SPI gel properties after PG treatment.

### 2.6. Molecular Forces in the Gels

**Figure 6:**
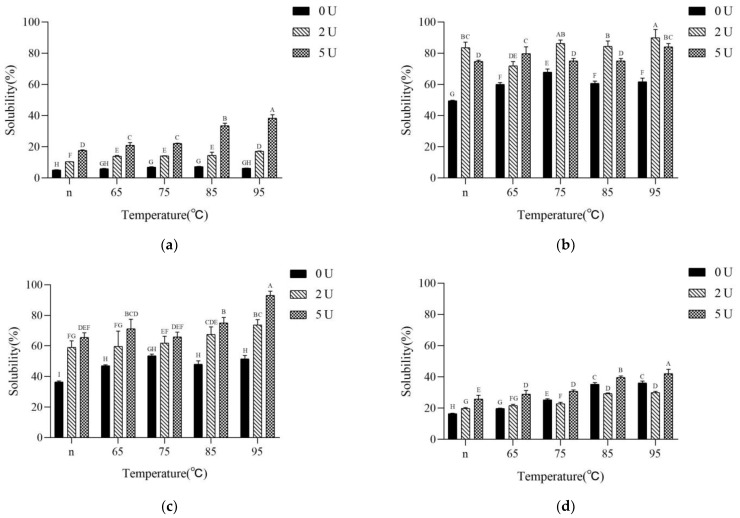
Solubility of SPI gels in different solvents. (**a**): 50 mM sodium phosphate (pH 7.0); (**b**): consisted of (**a**) and 8 M urea; (**c**): consisted of (**a**) and 0.5% SDS; (**d**): consisted of (**a**) and 0.25% βME. Different capital letters indicate significant differences (*p* < 0.05).

The interaction forces involved in gel network formation were evaluated by the relative ability of SPI gels to disperse in different solvents. The solubility of the CaSO_4_-induced gels in the phosphate buffer showed significant increase with the increase of PG addition (Figure 6a). However, it was worth noting that this increase in solubility was not proportional to the degree of deamidation of the sample itself. The sample treated with preheating at 95 °C and added enzyme in the amount of 5 U/g did not have high deamidation but high solubility. This result indicated that the number of proteins that could not be involved in the gel increased after deamidation.

Figure 6b showed that the solubility of CaSO_4_-induced gels in 8M urea was the highest in all pretreatment temperature systems for 2 U/g of PG-added samples. For 5 U/g of PG-added samples, the solubility was increased in native and lightly expanded samples. Samples with 7S fully denatured were lower than the samples without deamidation, indicating that the CaSO_4_-induced gel process hydrogen bonding of products increased in systems with slow deamidation, regardless of high or low deamidation, while the hydrogen bonding of the gel process was enhanced by light deamidation in systems with fast deamidation, while heavy deamidation was not conducive to hydrogen bonding.

Figure 6c showed that the hydrophobic interactions increased with the elevated heating temperature for the samples without deamidation. The deamidation more or less increased the solubility of CaSO_4_-induced gels in SDS, indicating that the hydrophobic interaction in the gel force was elevated after deamidation.

Figure 6d showed that for the samples without deamidation, the disulfide bond participation of the calcium-promoted gel process was significantly increased with the increase of heating temperature. In the CaSO_4_-induced gels after deamidation, the disulfide bond participation was significantly reduced compared with the samples without deamidation, and the lowest disulfide bond participation was observed for the samples with 5 U/g of enzyme addition after preheating treatment at 95 °C.

The correlation of the above results with the gel properties revealed that the increase of CaSO_4_-induced gel strength for the samples treated with preheating could be mainly due to the enhancement of hydrogen bonds, hydrophobic interactions and disulfide bonds during the gelation process, especially the significant increase of disulfide bonds, which should play a more important role. In contrast, the hydrophobic interactions were not weakened after SPI deamidation, the proportion of soluble protein was significantly increased, and the disulfide bonds were significantly weakened, which led to a partial decrease in gel strength and a partial increase in water-holding capacity. If the percentage of soluble protein in the gel system was further increased and the disulfide and hydrogen bonds were simultaneously weakened, the gel strength was further weakened (5 U/g of enzyme addition) and the water-holding capacity was further increased.

**Figure 7:**
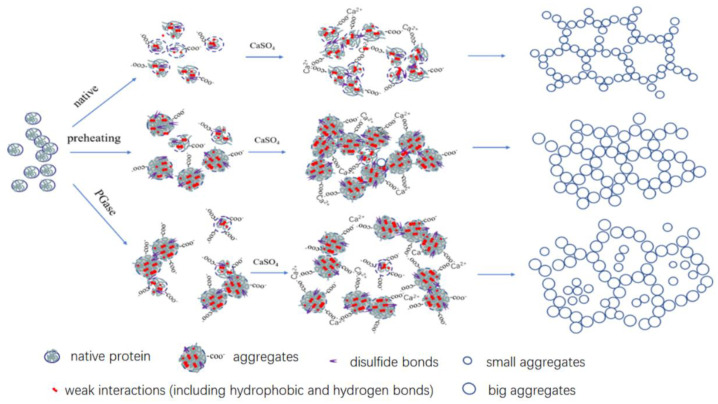
Supposed mechanism for the SPI with different heat treatment and deamidation levels on the gelation. Schematic: size and distance are not to scale.

The above results also further suggested that the mechanism of the effect of PG on the calcium-promoted gels of SPI with different preheating treatments was related to the amount and role of the molecules involved in the gel system after deamidation. If a large amount of protein was free in the gel network only as a filler component and was easily solubilized out by phosphate. This led to less protein forming the gel matrix, resulting in relatively low gel strength. These highly soluble proteins contributed positively to the water-holding capacity. In the case of the protein aggregates without deamidation treatment, the exposure of sulfhydryl groups was increased with increasing heat treatment intensity. As a result, more disulfide bonds were involved in gel formation, contributing to a significant increase in gel strength. The insufficient amount of soluble protein filling the gel network caused little change in the water-holding capacity of the gels. Due to deamidation, the large involvement of disulfide bonds in the preaggregation led to a decrease in the involvement of disulfide bonds formed during later CaSO_4_-induced gels, resulting in weaker gel strength. The carboxyl groups released by deamidation could slow down the calcium-promoted aggregation and enhance the water-holding capacity (Figure 7). Wang also pointed out that the increase in soluble protein content made the protein molecules less sensitive to Ca^2+^, which could lower the odds of direct interaction between Ca^2+^ and protein. This implies that excessive Ca^2+^ had more opportunities to interact with proteins. [44].

### 2.7. SEM

**Figure 8:**
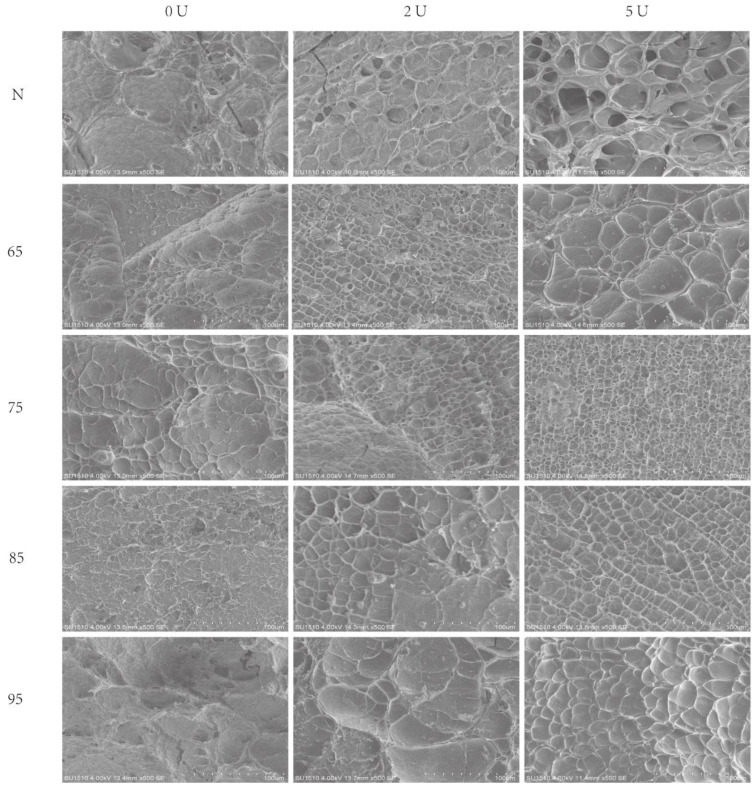
Scanning electron micrographs of SPI gels.

The SEM images showed the calcium-promoted gel structures of SPI with different degrees of thermal denaturation by PG treatment. Under the conditions without the addition of PG, the SPI gel structure was loose, and the heat treatment at 95 °C caused the protein structure to unfold and the gel network to be coarser (Figure 8).

After the addition of PG, the protein gels were dense and porous with the increase of PG addition, and the pores increased with the increase of PG addition, probably due to the deamidation of PG, which made the gels smoother and denser due to the increase of calcium bridge sites. As the heat treatment intensity increased, the SPI structure further unfolded, releasing more calcium bridge sites, which eventually led to a more homogeneous and detailed structure of the 95-5U gel sample and a stronger ability to hold moisture. Kao et al. found that gels with a homogeneous, ordered structure had higher water-holding capacity than those with a disordered gel structure [26].

## 3. Materials and Methods

### 3.1. Materials

Soybeans (626) were purchased from Fengyuan Zhongye Co., Ltd. (Shenyang, China) Glutaminase (500 U/g) was purchased from Amano Enzyme, Inc. (Nagoya, Japan) and stored at 4 °C. Ammonia assay kits were purchased from Sigma-Aldrich Co. (St. Louis, MO, USA). All aqueous solutions were prepared using deionized water. All other reagents and solvents were of at least analytical grade.

### 3.2. Preparation of Native Soy Protein Isolate

Native soy protein isolate (NSPI) was prepared from soybeans according to the procedure [45]. First, Soybeans were milled and defatted with a threefold concentration of n-hexane: ethyl alcohol (9:1, *v/v*). The defatted soy flour was dispersed in deionized water (1:10, *w/v*) at pH 8.0 while stirring for 2 h. The mixture was centrifuged at 10,000× *g* for 20 min at 4 °C. The supernatant was adjusted to pH 4.5 and allowed to stand for 30 min followed by being centrifuged (3300× *g*, 10 min). The precipitate was redissolved in deionized water at a ratio of 1:2 (*w/v*) for at least 3 h while maintaining a pH of 7.0. The solution was lyophilized to yield SPI with a protein content of 89.99%, as determined by the Kjeldahl method. The SPI was stored at −20 °C prior to use.

### 3.3. Preparation of Preheated SPI and Enzymatic Deamidation Products and Their CaSO_4_-Induced Gel Products

NSPI (7%) was subjected to different degrees of heat treatment at 65, 75, 85 and 95 °C for 10 min. After cooling to room temperature, 2 U/g and 5 U/g of PG were added to the samples at 37 °C for 1 h. The deamidated protein samples were named as N-0U, N-2U, N-5U, 65-0U, 65-2U, 65-5U, 75-0U, 75-2U, 75-5U, 85-0U, 85-2U, 85-5U, 95-0U, 95-2U, 95-5U.

The above sample solutions were heated to 95 °C for enzyme inactivation treatment, cooled to 85 °C and added CaSO_4_ (35 mM). After coagulation, the gels were cooled to room temperature and stored at 4 °C.

### 3.4. Determining the Degree of Deamidation

Amounts of ammonia released were measured using an ammonia test kit (Sigma). The degree of deamidation was expressed as the ratio of the amount of ammonia released by the PG reaction and the total glutamine residues of proteins. The total ammonia released from the raw protein samples was determined by preparing a 5% of soybean isolate solution with 2 M HCl and hydrolyzing it at 110 °C for 4 h [35]. 

### 3.5. Particle Size Analysis

0.5% (*w/v*) SPI dispersions with deionized water were prepared. The particle size was determined using a Zetasizer NanoZS instrument at 25 °C. 

### 3.6. Surface Hydrophobicity (S_0_)

ANS was used as a fluorescent probe to determine the S_0_ values of proteins. The protein samples were firstly configured with 0.01 mol/L phosphate buffer (pH 7.0) to a series of concentrations of 0.2, 0.4, 0.6, 0.8 and 1 mg/mL and then 20 μL of 8 mmol/L ANS solution was added to 4 mL of the above concentrations of protein solution, shaken well and left for 3 min, and then the fluorescence intensity was recorded at an excitation wavelength of 390 nm and an emission wavelength of 470 nm by using an F-2700 spectrofluorometer (Hitachi, Japan). The slope of the curve is the surface hydrophobicity value of the protein.

### 3.7. Measurement of Rheological Properties

Immediately after adding CaSO_4_, the SPI dispersion was transferred to the button plate of the rheometer. Silicone oil was added around the perimeter to prevent moisture loss. The gels oscillated at 1% strain (within the linear viscoelastic region, LVR) and a frequency of 1 Hz, the temperature was heated from 25 °C to 90 °C at 5 °C per minute, then down to 85 °C at 5 °C per minute, followed by incubation at this temperature for 10 min before cooling to 25 °C at 5 °C per minute. The storage modulus (G′), and loss modulus (G″), were recorded.

### 3.8. Gel Strength and Water-Holding Capacity

The gel strength of the samples was determined using a TA-XT Plus texture analyzer. A T/0.5 cylindrical probe (12.7 mm diameter) was used to compress the gel sample in the beaker (with volume of 25 mL) at a rate of 0.5 mm/s. The gel strength was defined as the maximum force (g) required to break the gel.

Protein gels were carefully removed from the beaker, weighed and transferred into 50 mL centrifuge tubes and centrifuged at 10,000 g for 15 min. The water stains on the surface of the gels after centrifugation were carefully removed using filter paper. The water-holding capacity (%) was defined as the percentage of gel mass before and after centrifugation multiplied by 100.

### 3.9. Scanning Electron Microscopy (SEM)

Small pieces of gel were removed from gel samples and immersed in 2.5% glutaraldehyde for 12 h. Samples were rapidly frozen in liquid nitrogen (−196 °C) and then freeze-dried. The microstructure of the samples was observed under a SU1510 electron microscope with gold spray and under an accelerating voltage of 4 kV.

### 3.10. Molecular Forces in the Gels

1 g gel samples were mixed with 9 mL of solvents using a Model Ultra-Turrax18 homogenizer. Solvent A was: 50 mM sodium phosphate (pH 7.0); B consisted of A and 8 M urea; C consisted of A and 0.5% SDS; D consisted of A and 0.25% βME. The homogenates were centrifuged at 10,000 g for 15 min. The amount of protein extracted after treatment with different solvents was used to express the main forces acting in the gel.

### 3.11. Statistical Analysis

All the experiments were performed in triplicate using freshly prepared solvents unless specified otherwise. Results are given as means ± standard deviation (SD) using the general linear model procedure in the Statistix software 9.0 (Analytical Software, Tallahassee, FL, USA). Significance was considered at *p* < 0.05.

## 4. Conclusions

PG deamidation of preheated SPI improved their water-holding capacity of gels. The best water-holding capacity of gel was obtained with 95 °C and 5 U/g of PG treatment. On the one hand, as the heat treatment intensity increased, the hydrogen bonding, hydrophobic interactions and disulfide bonding of the gel gelation process were all enhanced, making the gel strength gradually increase. On the other hand, as the concentration of PG increased, the percentage of soluble protein increased without significantly weakening hydrophobicity, resulting in a significant increase in water-holding capacity, while the disulfide bond was significantly weakened, resulting in a significant decrease in gel strength. In addition, the release of carboxyl groups from deamidation could also slow down the aggregation of calcium and improve water-holding capacity.

## Figures and Tables

**Table 1:** The end-point elastic modulus (G′) of the gel and the gel temperature (T_gel_ *).

Amount of Enzyme Addition	0U		2U		5U	
G′ and T_gel_	G′ (Pa)	T_gel_ (°C)	G′ (Pa)	T_gel_ (°C)	G′ (Pa)	T_gel_ (°C)
native	757.69	81.15	267.71	85.33	132.50	89.60
65	1360.15	72.26	831.85	83.03	155.48	89.54
75	2944.65	57.18	2897.44	80.74	1090.23	85.23
85	8550.76	26.89	5180.77	44.12	1313.29	78.73
95	13,503.27	42.24	6960.80	40.02	4512.01	50.71

* The temperature at which G′ started to increase over 0.5 Pa/K was defined as the gelation temperature [41].

## Data Availability

The data presented in this study are available on request from the corresponding author.

## Sample Availability

Samples of the compounds are not available from the authors.

## References

[1] Lu X., Lu Z., Yin L., Cheng Y., Li L. (2010). Effect of Preheating Temperature and Calcium Ions on the Properties of Cold-Set Soybean Protein Gel. Food Res. Int..

[2] Van Vliet T., Martin A.H., Bos M.A. (2002). Gelation and Interfacial Behaviour of Vegetable Proteins. Curr. Opin. Colloid Interface Sci..

[3] Ramírez-Suárez J.C., Xiong Y.L. (2003). Effect of Transglutaminase-Induced Cross-Linking on Gelation of Myofibrillar/Soy Protein Mixtures. Meat Sci..

[4] Sha L., Xiong Y.L. (2020). Trends in Food Science & Technology Plant Protein-Based Alternatives of Reconstructed Meat: Science, Technology, and Challenges. Trends Food Sci. Technol..

[5] Lu Z., Liu Y., En Y., Lee J., Chan A., Lee P., Yang H. (2023). Effect of Starch Addition on the Physicochemical Properties, Molecular Interactions, Structures, and in Vitro Digestibility of the Plant-Based Egg Analogues. Food Chem..

[6] Yan J., Yin L., Qu Y., Yan W., Zhang M., Su J., Jia X. (2022). Effect of Calcium Ions Concentration on the Properties and Microstructures of Doubly Induced Sorghum Arabinoxylan/Soy Protein Isolate Mixed Gels. Food Hydrocoll..

[7] Tang C.H., Li L., Wang J.L., Yang X.Q. (2007). Formation and Rheological Properties of “cold-Set” Tofu Induced by Microbial Transglutaminase. LWT.

[8] Zheng L., Teng F., Wang N., Zhang X.N., Regenstein J.M., Liu J.S., Li Y., Wang Z.J. (2019). Addition of Salt Ions before Spraying Improves Heatand Cold-Induced Gel Properties of Soy Protein Isolate (SPI). Appl. Sci..

[9] Alting A.C., De Jongh H.H.J., Visschers R.W., Simons J.W.F.A. (2002). Physical and Chemical Interactions in Cold Gelation of Food Proteins. J. Agric. Food Chem..

[10] Maltais A., Remondetto G.E., Gonzalez R., Subirade M. (2005). Formation of Soy Protein Isolate Cold-Set Gels: Protein and Salt Effects. J. Food Sci..

[11] Prabhakaran M.P., Perera C.O., Valiyaveettil S. (2006). Effect of Different Coagulants on the Isoflavone Levels and Physical Properties of Prepared Firm Tofu. Food Chem..

[12] Skurray G., Cunich J., Carter O. (1980). The Effect of Different Varieties of Soybean and Calcium Ion Concentration on the Quality of Tofu. Food Chem..

[13] Wang X., Luo K., Liu S., Adhikari B., Chen J. (2019). Improvement of Gelation Properties of Soy Protein Isolate Emulsion Induced by Calcium Cooperated with Magnesium. J. Food Eng..

[14] Min S., Yu Y., St. Martin S. (2005). Effect of Soybean Varieties and Growing Locations on the Physical and Chemical Properties of Soymilk and Tofu. J. Food Sci..

[15] Zhao H., Li W., Qin F., Chen J. (2016). Calcium Sulphate-Induced Soya Bean Protein Tofu-Type Gels: Influence of Denaturation and Particle Size. Int. J. Food Sci. Technol..

[16] Puppo M.C., Añón M.C. (1998). Structural Properties of Heat-Induced Soy Protein Gels as Affected by Ionic Strength and PH. J. Agric. Food Chem..

[17] Jian H., Qiao F., Yang P., Guo F., Huang X., Adhikari B., Chen J. (2016). Roles of Soluble and Insoluble Aggregates Induced by Soy Protein Processing in the Gelation of Myofibrillar Protein. Int. J. Food Sci. Technol..

[18] Zhao C., Yin H., Yan J., Niu X., Qi B., Liu J. (2021). Structure and Acid-Induced Gelation Properties of Soy Protein Isolate–Maltodextrin Glycation Conjugates with Ultrasonic Pretreatment. Food Hydrocoll..

[19] Tang C.H., Yang M., Liu F., Chen Z. (2013). Stirring Greatly Improves Transglutaminase-Induced Gelation of Soy Protein-Stabilized Emulsions. LWT.

[20] Hu H., Li-Chan E.C.Y., Wan L., Tian M., Pan S. (2013). The Effect of High Intensity Ultrasonic Pre-Treatment on the Properties of Soybean Protein Isolate Gel Induced by Calcium Sulfate. Food Hydrocoll..

[21] Guo S.T., Ono T. (2005). The Role of Composition and Content of Protein Particles in Soymilk on Tofu Curding by Glucono-δ-Lactone or Calcium Sulfate. J. Food Sci..

[22] Nicole M., Caimeng Z., Joseph H., Eric K., Yufei H. (2016). Soybean Oil Volume Fraction Effects on the Rheology Characteristics and Gelation Behavior of Glucono-δ-Lactone and Calcium Sulfate-Induced Tofu Gels. J. Texture Stud..

[23] Brito-Oliveira T.C., Cavini A.C.M., Ferreira L.S., Moraes I.C.F., Pinho S.C. (2020). Microstructural and Rheological Characterization of NaCl-Induced Gels of Soy Protein Isolate and the Effects of Incorporating Different Galactomannans. Food Struct..

[24] Wu M., Xiong Y.L., Chen J., Tang X., Zhou G. (2009). Rheological and Microstructural Properties of Porcine Myofibrillar Protein-Lipid Emulsion Composite Gels. J. Food Sci..

[25] Nicole M., Caimeng Z., Eric K., Yufei H. (2014). Salt and Acid-Induced Soft Tofu-Type Gels: Rheology, Structure and Fractal Analysis of Viscoelastic Properties as a Function of Coagulant Concentration. Int. J. Food Eng..

[26] Kao F.J., Su N.W., Lee M.H. (2003). Effect of Calcium Sulfate Concentration in Soymilk on the Microstructure of Firm Tofu and the Protein Constitutions in Tofu Whey. J. Agric. Food Chem..

[27] Ziegler G.R. (1990). The Gelation of Proteins. Adv. Food Nutr. Res..

[28] Urbonaite V., de Jongh H.H.J., van der Linden E., Pouvreau L. (2015). Water Holding of Soy Protein Gels Is Set by Coarseness, Modulated by Calcium Binding, Rather than Gel Stiffness. Food Hydrocoll..

[29] Wang X., He Z., Zeng M., Qin F., Adhikari B., Chen J. (2017). Effects of the Size and Content of Protein Aggregates on the Rheological and Structural Properties of Soy Protein Isolate Emulsion Gels Induced by CaSO4. Food Chem..

[30] Tang C.H., Chen L., Foegeding E.A. (2011). Mechanical and Water-Holding Properties and Microstructures of Soy Protein Isolate Emulsion Gels Induced by CaCl_2_, Glucono-δ-Lactone (GDL), and Transglutaminase: Influence of Thermal Treatments before and/or after Emulsification. J. Agric. Food Chem..

[31] Miwa N., Yokoyama K., Wakabayashi H., Nio N. (2010). Effect of Deamidation by Protein-Glutaminase on Physicochemical and Functional Properties of Skim Milk. Int. Dairy J..

[32] Jiang Y., Wang Z., He Z., Zeng M., Qin F., Chen J. (2022). Effect of Heat-Induced Aggregation of Soy Protein Isolate on Protein-Glutaminase Deamidation and the Emulsifying Properties of Deamidated Products. LWT.

[33] Yamaguchi S., Yokoe M. (2000). A Novel Protein-Deamidating Enzyme from Chryseobacterium Proteolyticum Sp. Nov., a Newly Isolated Bacterium from Soil. Appl. Environ. Microbiol..

[34] Jiang Z.Q., Sontag-Strohm T., Salovaara H., Sibakov J., Kanerva P., Loponen J. (2015). Oat Protein Solubility and Emulsion Properties Improved by Enzymatic Deamidation. J. Cereal Sci..

[35] Suppavorasatit I., De Mejia E.G., Cadwallader K.R. (2011). Optimization of the Enzymatic Deamidation of Soy Protein by Protein-Glutaminase and Its Effect on the Functional Properties of the Protein. J. Agric. Food Chem..

[36] Sorgentini D.A., Wagner J.R., Anon M.C. (1995). Effects of Thermal Treatment of Soy Protein Isolate on the Characteristics and Structure-Function Relationship of Soluble and Insoluble Fractions. J. Agric. Food Chem..

[37] Watanabe T. (1979). Influence of Heating Temperature on Conformational Changes of Soybean Proteins. Agric. Biol. Chem..

[38] Yie H.Y., Yamaguchi S., Yeun S.G., Mori T., Matsumura Y. (2004). Effects of Enzymatic Deamidation by Protein-Glutaminase on Structure and Functional Properties of α-Zein. J. Agric. Food Chem..

[39] Zhao J., Tian Z., Chen L. (2011). Effects of Deamidation on Aggregation and Emulsifying Properties of Barley Glutelin. Food Chem..

[40] Mao L., Roos Y.H., Miao S. (2014). Study on the Rheological Properties and Volatile Release of Cold-Set Emulsion-Filled Protein Gels. J. Agric. Food Chem..

[41] Zhou J.Z., Zhang H., Gao L., Wang L., Qian H.F. (2015). Influence of PH and Ionic Strength on Heat-Induced Formation and Rheological Properties of Cottonseed Protein Gels. Food Bioprod. Process..

[42] Saio K., Koyama E., Yamazaki S., Watanabe T. (1969). Protein-Calcium-Phytic Acid Relationships in Soybean: Part III. Effect of Phytic Acid on Coagulative Reaction in Tofu-Making. Agric. Biol. Chem..

[43] Zhang G., Ma S., Liu X., Yin X., Liu S., Zhou J., Du G. (2021). Protein-Glutaminase: Research Progress and Prospect in Food Manufacturing. Food Biosci..

[44] Wang R., Xie L., Guo S. (2015). Effects of Small Molecular Compounds in Soymilk on the Protein Coagulation Process: Ca^2+^ as Coagulant. Food Res. Int..

[45] Feng J., Xiong Y.L. (2003). Interaction and Functionality of Mixed Myofibrillar and Enzyme-Hydrolyzed Soy Proteins. J. Food Sci..

